# Effects of interportal error on dose distribution in patients undergoing breath‐holding intensity‐modulated radiotherapy for pancreatic cancer: evaluation of a new treatment planning method

**DOI:** 10.1120/jacmp.v14i5.4252

**Published:** 2013-09-06

**Authors:** Toru Takakura, Mitsuhiro Nakamura, Keiko Shibuya, Manabu Nakata, Akira Nakamura, Matsuo Yukinori, Takeshi Shiinoki, Kyoji Higashimura, Teruki Teshima, Masahiro Hiraoka

**Affiliations:** ^1^ Clinical Radiology Service Division Kyoto University Hospital Kyoto; ^2^ Department of Medical Physics and Engineering Graduate School of Medicine Osaka University Osaka; ^3^ Department of Radiation Oncology and Image‐applied Therapy Graduate School of Medicine Kyoto University Kyoto; ^4^ Department of Therapeutic Radiology Graduate School of Medicine Yamaguchi University Yamaguchi; ^5^ Department of Radiation Oncology Osaka Medical Center for Cancer and Cardiovascular Diseases Osaka Japan

**Keywords:** IMRT, pancreas cancer, breath‐holding technique

## Abstract

In patients with pancreatic cancer, intensity‐modulated radiotherapy (IMRT) under breath holding facilitates concentration of the radiation dose in the tumor, while sparing the neighboring organs at risk and minimizing interplay effects between movement of the multileaf collimator and motion of the internal structures. Although the breath‐holding technique provides high interportal reproducibility of target position, dosimetric errors caused by interportal breath‐holding positional error have not been reported. Here, we investigated the effects of interportal breath‐holding positional errors on IMRT dose distribution by incorporating interportal positional error into the original treatment plan, using random numbers in ten patients treated for pancreatic cancer. We also developed a treatment planning technique that shortens breath‐holding time without increasing dosimetric quality assurance workload. The key feature of our proposed method is performance of dose calculation using the same optimized fluence map as the original plan, after dose per fraction in the original plan was cut in half and the number of fractions was doubled. Results confirmed that interportal error had a negligible effect on dose distribution over multiple fractions. Variations in the homogeneity index and the dose delivered to 98%, 2%, and 50% of the volume for the planning target volume, and the dose delivered to 1 cc of the volume for the duodenum and stomach were ± 1%, on average, in comparison with the original plan. The new treatment planning method decreased breath‐holding time by 33%, and differences in dose‐volume metrics between the original and the new treatment plans were within ± 1%. An additional advantage of our proposed method is that interportal errors can be better averaged out; thus, dose distribution in the proposed method may be closer to the planned dose distribution than with the original plans.

PACS number: 87.53.Bn, 87.55.D‐, 87.55.‐x

## I. INTRODUCTION

Pancreatic cancer is currently the fifth‐leading cause of death from cancer in Japan.^(1)^ The number of people dying from this cancer has increased annually, and reached approximately 28,000 deaths in 2010. Although the first‐choice curative treatment for pancreatic cancer remains surgery, more than 80% of patients have nonresectable disease at the time of diagnosis.^(2)^ These cases are often treated with chemoradiotherapy, but the presence of radiosensitive organs at risk (OARs) around the pancreas, including the duodenum and stomach, prevents the delivery of a sufficient radiation dose, which may result in unfavorable outcomes.^3^, ^4^, ^5^, ^6^, ^7^, ^8^, ^9^ Thus, an important issue in the treatment of pancreatic cancer generally is how to deliver a more intense radiation dose.

Intensity‐modulated radiotherapy (IMRT) facilitates the concentration of radiation dose in the tumor, while sparing doses to OARs, and can therefore reduce the rate of gastrointestinal toxicity.^10^, ^11^, ^12^ Respiratory motion remains an obstacle to dose delivery, however, and pancreatic tumor motion has been confirmed to be greater than 10 mm using several modalities.^(13)^ When respiratory motion is not managed, a larger internal margin is required to fully cover geometric changes in free breathing,^(14)^ which, in turn, results in the incorporation of a large volume of OARs into the planning target volume (PTV) and the possibility of severe gastrointestinal toxicity.^(15)^ Additionally, the dosimetric advantage of IMRT is degraded significantly by interplay between movement of the multileaf collimator (MLC) and motion of the internal structures,^16^, ^17^, ^18^ resulting in unintended underdose to the tumor and/or overdose to normal tissues. These problems seriously hamper the widespread adoption of IMRT for moving tumors, and accordingly indicate the need for respiratory management.

Our department is currently conducting a phase I/II radiation dose escalation study of full‐dose gemcitabine with IMRT in pancreatic cancer patients under end‐exhalation breath‐holding (EE‐BH) conditions with a visual‐feedback technique (BH‐IMRT).^16^, ^17^, ^18^ The goal is to evaluate the possible impact of our protocol on response, toxicity, pain relief, and outcome in patients with locally advanced nonresectable pancreatic cancer, with reference to previous dose escalation trials of full‐dose gemcitabine with conventional RT at the University of Michigan.^(^ ^9^ ^,^ ^19^ ^)^ We reported previously that the EE‐BH technique provided high interportal reproducibility of target position in pancreatic cancer.^(20)^ However, the effects of interportal BH positional error on dosimetric errors have not been reported before. Our previous study also showed that a minimum BH time of 15 sec was required at the lowest dose level (2.6 Gy per fraction) at a dose rate of 600 monitor units (MU)/min. MUs per port were increased at the higher prescription dose levels in dose‐escalation studies. A long BH time of > 15 sec at EE is typically not only difficult even for healthy people, but also has the potential to cause dosimetric error between the planned and delivered dose distribution as a result of baseline drift.^(21)^ Generally, MUs per port can be reduced in multiport plans having different gantry and couch angles, but dosimetric quality assurance (DQA) of multiport plans requires measurement of each port and is, thus, laborious and time consuming.

The purpose of the present study was to investigate the effects of interportal BH positional errors on dose distribution, and to propose a treatment planning technique that both reduces the effect of interportal BH positional errors and shortens BH time without increasing DQA workload.

## II. MATERIALS AND METHODS

This study was conducted in ten patients who underwent BH‐IMRT for pancreatic cancer at Kyoto University Hospital between May 2010 and June 2011. Clinical target volume (CTV) and OARs, including duodenum, stomach, kidney, liver, spleen, and spinal cord, were delineated manually by a single radiation oncologist to eliminate interobserver variation. A PTV was created by adding isotropic margins of 5 mm to the CTV. The dynamic IMRT plan was designed using Eclipse (Helios, ver. 8.6.15; Varian Medical Systems, Palo Alto, CA). Five fixed coplanar ports with gantry angles of 40°, 100°, 180°, 260°, and 320° were selected. The prescribed dose was 39 to 45 Gy in 15 fractions, with beam energy and dose rate of 15 MV photon beam and 600 MU/min, respectively. The treatment plan for the planning CT was used as the original treatment plan in the present study. The specification and technical details of CT data acquisition and dose constraints have been reported elsewhere.^20^, ^21^, ^22^

### A. Effects of interportal breath‐hold positional error on dose distribution

To incorporate interportal positional error into the treatment plan, a total of 750 sets of ten patients x five ports x 15 fractions, including LR, AP, and SI coordinates of random numbers were generated according to a normal distribution. Means and standard deviations (SDs) of the normal distribution were based on the results of our previous study.^(20)^ Each of the calculated random numbers was assigned to the isocenter position for each port, and doses were then recalculated under the same MUs and an identical beam setup. Variations in homogeneity index (HI) and the dose delivered to $98\%\;(D_{98\%}),2\%\;(D_{2\%})$, and $50\%\;(D_{50\%})$ of the volume for PTV, and the dose delivered to 1 cc of the volume $\left(D_{1\text{cc}}\right)$ for the duodenum and stomach were evaluated in comparison with the original plan. HI was calculated in accordance with the definition in ICRU report 83.^(23)^

### B. Treatment planning to shorten the breath‐hold time

**Figure 1:**
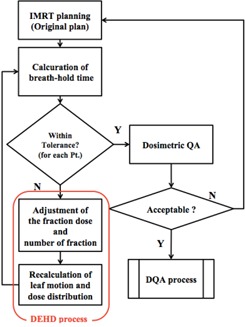
Flow chart of the DEHD planning procedure.

To shorten the BH time without complicating the DQA procedure, we propose a new treatment plan called the double‐exposure half‐dose plan (DEHD plan). First, the dose per fraction of the original plan was cut in half and the number of fractions was doubled, and leaf motion sequence was then determined using the same optimized fluence map as in the original plan. If this step is skipped, leaf motion speed is doubled, which results in the delay of MLC motion, due to exceeding the maximum leaf speed.^(^ ^24^ ^,^ ^25^ ^)^ Finally, the dose calculation was performed using newly created fluence maps. A flow chart for the procedure of the DEHD plan is shown in Fig. 1. The validity of this method was assessed by evaluating $\text{HI},\;D_{98\%},\;D_{50\%}$, and $D_{2\%}$ for the PTV, and $D_{1\text{cc}}$ for the stomach and duodenum.

### C. Comparison of calculated dose fluence maps

To verify the DEHD plan, the dose distributions for each port in the DEHD plan were calculated on a plane perpendicular to the radiation field at a depth of 10 cm with a virtual phantom on Eclipse, and were then compared with those in the original plan using commercially available radiation dosimetry software (DD system, ver. 9.4; R‐Tech Inc., Tokyo, Japan). The dose distribution in the DEHD plan was registered with that in the original plan, based on the isocenter. Dose distribution was not normalized, but was compared for the area receiving more than 50% of the isodose to evaluate the dose around the target using the dose difference criteria of 0.5%, 1.0%, and 2.0%, with a dose grid resolution of 0.39 mm.

## III. RESULTS & DISCUSSION

### A. Effects of interportal breath‐hold positional error on dose distribution

**Figure 2:**
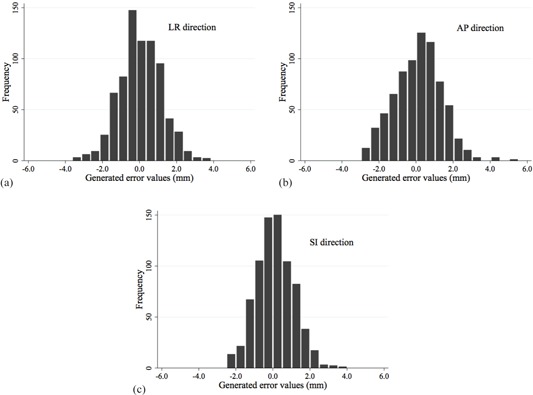
Histograms of error values generated using random numbers in the (a) LR, (b) AP, and (c) SI directions.

Frequency histograms of generated random numbers are shown in Fig. 2. Means ± SDs of random numbers were 0.07 ± 1.12 mm (range, ‐3.62 to 3.59 mm), 0.12 ± 0.99 mm (range, ‐2.47 to 3.54 mm), and 0.12 ± 1.26 mm (range, ‐2.95 to 5.33 mm) in LR, SI, and AP directions, respectively. These values were comparable to those reported previously.^(21)^

**Figure 3:**
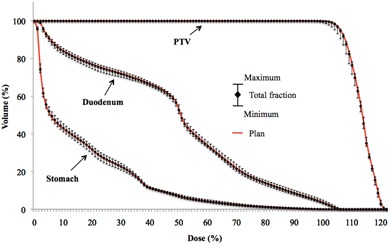
DVH for the case with the largest variation in HI for PTV.

Variations in $\text{HI},\;D_{98\%},\;D_{50\%}$, and $D_{2\%}$ for the PTV and $D_{1\text{cc}}$ for the stomach and duodenum are summarized in Table 1. The data in the third, fourth, and fifth columns are means ± SDs of dose volume metrics from ten patients in the original treatment plan, those from 150 fractions incorporating interportal positional error, and those from averaged sums of 15 fractions for each patient, respectively. The PTV was well‐covered by the planned dose, while $D_{1\text{cc}}$ for the stomach and duodenum varied interfractionally; however, these variations were small compared with the original plan. Figure 3 shows the dose‐volume histogram (DVH) for the case with the largest dosimetric variation in HI for PTV. The extent of the whiskers indicates the total range of variation from the original value for 15 fractions. Several investigators have indicated that the dosimetric deviations are averaged out over multiple fractions;^(^ ^26^ ^,^ ^27^ ^)^ on this basis, the effect of interportal variation on delivered dose after 15 fractions would seem to be negligible.

**Table 1:** Variations in dose‐volume metrics

*Structure*	*Parameter*	*Original*	*Each Fraction*	*Total Fraction*
PTV (%)	$D_{98\%}$	93.74±6.48	93.19±6.28	93.40±6.50
	$D_{2\%}$	110.10±5.94	110.09±5.65	110.97±5.93
	$D_{50\%}$	105.80±4.72	105.67±4.55	105.62±4.74
	HI	0.164±0.041	0.170±0.042	0.167±0.041
Duodenum (cGy/fr)	$D_{1\;\text{cc}}$	233.13–255.63	219.52–262.39	231.63–252.89
Stomach (cGy/fr)	$D_{1\;\text{cc}}$	43.32–256.14	34.45–259.73	45.53–255.86

PTV = planning target volume; $D_{\text{XX}\%}$ = dose covering a volume of XX%; HI = homogeneity index; $D_{1\text{cc}}$ = dose covering a volume of 1 cc.

### A. Evaluation of the DEHD plan

Table 2 summarizes the comparison of dosimetric parameters in $\text{HI},\;D_{98\%},\;D_{50\%}$, and $D_{2\%}$ by the mean ± SD in percentiles for the PTV and $D_{1\text{cc}}$ by the range in cGy for the stomach and duodenum, respectively. While the BH times required in the original plan ranged from 11.3 to 16.8 sec, those in the DEHD plan were in the range 7.9–11.4 sec. Among 50 ports, BH time was longer than 10 sec in only six (12%). The reduction in BH time was in the range 23.5%–40.3%.

**Figure 4:**
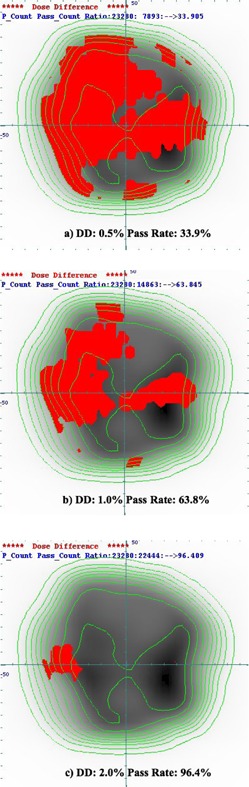
Comparison of dose‐difference maps between the original and DEHD plans for the case with the worst pass rate. The red areas indicate failure, with criteria of (a) 0.5%, (b) 1.0%, and (c) 2.0% for the area receiving more than 50% of the dose. The isodose lines displayed in the interval 10% are from the 10% to the 90% isodose lines.

**Figure 5:**
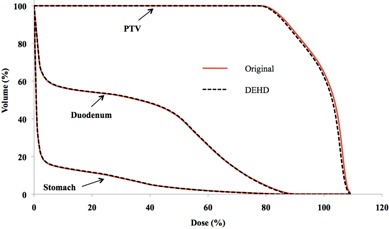
DVH of the original and DEHD plans for patient #7.

Means ± SDs of the pass rate of dose differences between the original and DEHD plans were 84.1% ± 14.6% (range, 33.9%–97.5%), 93.6% ± 9.9% (range, 58.1%–100.0%), and 97.9% ± 4.5% (range, 76.8%–100.0%), with criteria of 0.5%, 1.0%, and 2.0%, respectively (Table 3). The pass rate for the dose differences between the original and DEHD plans was generally high in 0.5% and 1.0%, except for patient #7. Figure 4 shows the dose difference map for the port having the worst pass rate (patient #7, port 4). Even when there were large dose differences between the original and DEHD plans, however, the dosimetric parameters in the DEHD plan were almost identical to those in the original plan (Fig. 5). The reason why large dose differences were observed may be that the MLC control points increased in the DEHD plan when recalculating the leaf motion and actual fluence map. Compared with other ports (31.1% on average), a marked increase in the MLC control points was observed for port 4 in patient #7 (39.8%), which may have caused the relatively large dose difference in actual fluence between the original and DEHD plan. In DMLC IMRT, breath‐holding time was not prolonged, even when the MLC control points were increased.

**Table 2:** Comparison of dose‐volume metrics between the original and DEHD plans

*Structure*	*Parameter*	*Original*	*DEHD*
PTV (%)	$D_{98\%}$	93.74±6.48	93.50±6.57
	$D_{2\%}$	110.10±5.94	110.96±6.00
	$D_{50\%}$	105.80±4.72	105.58±4.77
	HI	0.164±0.041	0.165±0.042
Duodenum (cGy/fr)	$D_{1\;\text{cc}}$	233.13–255.63	233.30–254.10
Stomach (cGy/fr)	$D_{1\;\text{cc}}$	43.32–256.14	44.30–255.64

PTV = planning target volume; $D_{\text{XX}\%}$ = dose covering a volume of XX%; HI = homogeneity index; $D_{1\text{cc}}$ = dose covering a volume of 1 cc.

**Table 3:** Pass rate of dose differences between the original and DEHD plans for each patient

*Threshold*		*Pt. 1*	*Pt. 2*	*Pt. 3*	*Pt. 4*	*Pt. 5*	*Pt. 6*	*Pt. 7*	*Pt. 8*	*Pt. 9*	*Pt. 10*
0.5%	Mean (%)	83.1	95.4	86.9	88.3	86.3	87.3	52.0	80.6	90.2	91.0
	SD (%)	2.5	1.4	6.0	5.5	9.4	8.7	20.1	15.8	5.4	6.0
1.0%	Mean (%)	93.8	99.6	94.4	98.8	94.7	97.2	74.2	88.4	97.0	97.9
	SD (%)	2.9	0.4	4.0	2.2	6.5	3.2	17.1	13.3	1.8	2.6
2.0%	Mean (%)	98.4	100.0	97.3	100.0	98.1	99.9	92.7	93.7	99.4	99.9
	SD (%)	2.0	0.0	1.8	0.1	2.1	0.1	8.0	9.6	0.8	0.3

SD = standard deviation.

The DEHD plan was capable of reducing BH time by 33%, on average, without markedly reducing the dose‐volume parameters in the original plan, facilitating the treatment of patients who find prolonged BH difficult. However, this method increases the frequency of breath holding and prolongs the time the patient is required to maintain the same posture. Accordingly, it is desirable to use the DEHD plan only when difficulties in breath‐hold time are expected, or large dosimetric errors in the patient's body are predicted due to poor reproducibility of the breath‐holding position. Selection of the plan in consideration of these advantages and disadvantages can reduce the physical distress in patients and deviations of the actual dose distribution from that calculated in the treatment plan. When the number of ports increased from the original plan, BH time was shortened, but dose‐volume metrics and dose distributions were sometimes different from the original plan. The DEHD plan uses the optimized fluence map of the original plan; thus, further optimization processes are not needed once dose‐volume constraints in the original plan are satisfied, while radiation oncologists using the DEHD plan must verify dose‐volume metrics and dose distributions. Additionally, medical physicists must check the machine condition and parameters in the radiation treatment planning system and perform additional DQA if there are large differences between the calculated and measured doses. An additional advantage of our proposed method is that interportal BH positional errors can be better averaged out. The dose distribution in the DEHD plan can thus be closer to the planned dose distribution than with the original plan.

## IV. CONCLUSIONS

We demonstrated that the effects of interportal error on dose distribution in BH‐IMRT are negligible. Additionally, we propose a new method of treatment planning, called the “DEHD plan”. The DEHD plan can shorten BH time without substantially reducing dose‐volume metrics and without increasing DQA workload, compared with that required for a multiport plan, because only one of the two identical beams is measured. Finally, the effects of interportal error on dose distribution can be reduced through using the DEHD plan.

## ACKNOWLEDGMENTS

This work was supported by a Grant‐in‐Aid for the Encouragement of Scientists from the Ministry of Education, Culture, Sports, Science and Technology, Japan (Grant No. 23931024), and a Grant‐in‐Aid for Young Scientists (B) from the Ministry of Education, Culture, Sports, Science and Technology, Japan (Grant No. 23791408).
